# Parental inheritance and perinatal tobacco smoke exposure increase the gender-dependent risk of physician diagnosed asthma at preschool age

**DOI:** 10.1186/1710-1492-10-S1-A54

**Published:** 2014-03-03

**Authors:** Chih-Chiang Wu, Te-Yao Hsu, Ho-Chang Kuo, Chia-Yu Ou, Jen-Chieh Chang, Chieh-An Liu, Chih-Lu Wang, Hua Chuang, Hsiu-Mei Liang, Kuender D Yang

**Affiliations:** 1 Department of Pediatrics, Show Chwan Memorial Hospital, ChangHua, Taiwan; 2 Department of Medical Research, Show Chwan Health Care System in Chang Bing, Changhua, Taiwan; 3 Institute of Clinical Medicine, National Yang-Ming University, Taiwan; 4 Department of Obstetrics and Gynecology, Kaohsiung Chang Gung Memorial Hospital, Taiwan and Chang Gung University College of Medicine, Kaohsiung, Taiwan; 5 Department of Pediatrics, Kaohsiung Chang Gung Memorial Hospital and Chang Gung University College of Medicine, Kaohsiung, Taiwan; 6 Division of Dermatology, Department of Medicine, McGill University Health Centre, CanadaInstitute of Biomedical Sciences, National Sun Yat-Sen University, Kaohsiung, Taiwan; 7 Genomic and Proteomic Core Laboratory, Department of Medical Research, Kaohsiung Chang Gung Memorial Hospital and Chang Gung University College of Medicine, Kaohsiung, Taiwan; 8 Department of Pediatrics, Po-Jen Hospital, Kaohsiung, Taiwan; 9 Department of Pediatrics, Po-Jen Hospital, Kaohsiung, Taiwan

## Background

Genetic inheritance and perinatal tobacco smoke exposure (TSE) have been proven to be critical for the development of childhood allergic diseases [1, 2]. This study investigated the interactive roles of parental allergic histories and TSE on the development of childhood asthma at 6 years old.

## Methods

A birth cohort in southern Taiwan was studied. Information about parental allergic histories, gender, prematurity, TSE, and childhood allergic disease ever diagnosed by a physician were acquired from questionnaire during follow up. Children were asked to follow up at 6 years of age for allergic questionnaire and sensitization examination (CAP system).

## Results

In this cohort study, 748 of the children with complete data were analyzed. 217 (29%) of children had positive parental allergic history, 191 (25.5%) of children had TSE history, and 186 (24.9%) of children had been diagnosed as asthma by a physician in the first 6 years of life. In a regression analysis, physician diagnosed asthma ever in the first 6 years of life were significantly associated with male gender (OR: 1.941, 95% CI: 1.371-2.748, p<0.001), either parent with allergic diseases (OR: 1.548, 95% CI: 1.047-2.288, p=0.028), and TSE (OR: 1.504, 95% CI: 1.038-2.179, p=0.031), but not significantly associated with preterm (p=0.801). TSE with more than 20 cigarettes per day made children significantly higher risky to have physician-diagnosed-asthma than those with smoke exposure less than 20 cigarettes per day or those without smoke exposure (35%, 25% and 22.7% respectively, p=0.003). TSE was not related to physician diagnosed rhinitis, dermatitis or allergic sensitization by 6 years of age (p>0.5). Besides, TSE and parental allergic history had synergistic influence on the physician diagnosed asthma ever in the 6 years of life. This synergistic influence was significant in girls, rather than in boys (Table 1).

**Table 1:** TSE and parental allergic history had synergistic influence on the physician diagnosed asthma ever in the 6 years of life. This synergistic influence was significant in girls, rather than boys

All	physician diagnosed asthma		OR	95%CI	p (compared with A)
parent allergic disorder -, TSE- (A)	30/161	18.60%	1		
parent allergic disorder +, TSE-	97/396	24.00%	1.417	0.896-2.240	0.135
parent allergic disorder -, TSE+	13/56	23.20%	1.32	0.632-2.757	0.459
parent allergic disorder +, TSE+	46/135	34.10%	2.257	1.325-3.846	0.002

Girls					p (compared with A1)

parent allergic disorder -, TSE- (A1)	4/72	5.60%	1		
parent allergic disorder +, TSE-	38/196	19.40%	4.089	1.404-11.905	0.006
parent allergic disorder -, TSE+	3/23	13.00%	2.55	0.526-12.353	0.231
parent allergic disorder +, TSE+	20/61	32.80%	8.293	2.649-25.964	<0.001

Boys					p (compared with A2)

parent allergic disorder -, TSE- (A2)	28/89	29.20%	1		
parent allergic disorder +, TSE-	59/200	29.50%	1.014	0.586-1.755	0.961
parent allergic disorder -, TSE+	10/33	30.30%	1.054	0.441-2.519	0.907
parent allergic disorder +, TSE+	26/74	35.10%	1.313	0.678-2.541	0.419

## Conclusions

In the prospective cohort study, we found that male gender, parental allergic history, and TSE were significantly associated with physician diagnosed asthma by 6 years of age. TSE and parental allergic history had synergistic effect on the physician diagnosed asthma by 6 years of age. This synergistic influence was significant in girls, rather than boys.

